# Designing and Testing of a System for Aerosolization and Recovery of Viable Porcine Reproductive and Respiratory Syndrome Virus (PRRSV): Theoretical and Engineering Considerations

**DOI:** 10.3389/fbioe.2021.659609

**Published:** 2021-05-10

**Authors:** Peiyang Li, Jacek A. Koziel, Jeffrey J. Zimmerman, Steven J. Hoff, Jianqiang Zhang, Ting-Yu Cheng, Wannarat Yim-Im, Myeongseong Lee, Baitong Chen, William S. Jenks

**Affiliations:** ^1^Department of Agricultural and Biosystems Engineering, Iowa State University, Ames, IA, United States; ^2^Department of Veterinary Diagnostic and Production Animal Medicine, Iowa State University, Ames, IA, United States; ^3^Department of Chemistry, Iowa State University, Ames, IA, United States

**Keywords:** airborne pathogens, animal production, infectious animal disease, livestock health, mass balance, swine diseases, viral aerosol, virus isolation

## Abstract

Porcine reproductive and respiratory syndrome virus (PRRSV) infections cause significant economic losses to swine producers every year. Aerosols containing infectious PRRSV are an important route of transmission, and proper treatment of air could mitigate the airborne spread of the virus within and between barns. Previous bioaerosol studies focused on the microbiology of PRRSV aerosols; thus, the current study addressed the engineering aspects of virus aerosolization and collection. Specific objectives were to (1) build and test a virus aerosolization system, (2) achieve a uniform and repeatable aerosol generation and collection throughout all replicates, (3) identify and minimize sources of variation, and (4) verify that the collection system (impingers) performed similarly. The system for virus aerosolization was built and tested (Obj. 1). The uniform airflow distribution was confirmed using a physical tracer (<12% relative standard deviation) for all treatments and sound engineering control of flow rates (Obj. 2). Theoretical uncertainty analyses and mass balance calculations showed <3% loss of air mass flow rate between the inlet and outlet (Obj. 3). A comparison of TCID_50_ values among impinger fluids showed no statistical difference between any two of the three trials (*p*-value = 0.148, 0.357, 0.846) (Obj. 4). These results showed that the readiness of the system for research on virus aerosolization and treatment (e.g., by ultraviolet light), as well as its potential use for research on other types of airborne pathogens and their mitigation on a laboratory scale.

## Introduction

Porcine reproductive and respiratory syndrome (PRRS) is one of the most economically impactful diseases that need to be mitigated to ensure animal production security. The annual cost of PRRS to producers was estimated to be $560 million estimated in 2005 (Neumann et al., 2005), $664 million in 2012 (Holtkamp et al., 2013), and $580 million in 2016 (Miller, 2017). The disease is caused by a single-stranded RNA virus (PRRSV), initially described by Terpstra et al. (1991) and Wensvoort et al. (1991).

Air and aerosols are an important route of transmission for some infectious diseases, e.g., foot-and-mouth disease virus (FMDV) (Donaldson and Alexandersen, 2002) and influenza virus (Herfst et al., 2012). PRRSV can be transmitted *via* indirect contact (such as aerosol and fomites) or direct contact, but it likewise is found in aerosols generated by infected pigs and reach susceptible pigs meters or kilometers away (Torremorell et al., 1997; Wills et al., 1997; Lager and Mengeling, 2000; Otake et al., 2002; Dee et al., 2005; Arruda et al., 2019). Indeed, research suggested that aerosols of infectious PRRSV can travel up to ∼9 km (Dee et al., 2009; Otake et al., 2010). The meteorological conditions that favored long-distance transmission of airborne virus included low temperature, moderate levels of relative humidity, rising barometric pressure, low wind speed, and low sunlight intensity (Dee et al., 2010). It was reported that PRRSV could be infectious for 24 h at 37° C (or 99F∘) and survive for 6 days at 21°C (or 70F∘) (Pitkin et al., 2017). Given its airborne features and survivability, proper treatment of PRRSV aerosols could effectively reduce the transmission of the disease.

Previous research on the aerosolized PRRSV focused primarily on virus detection and secondarily on engineering, e.g., control of flow rate, pressure, mass balance, and uncertainty analyses. Hermann et al. (2006) optimized a sampling system with six channels for simultaneous PRRSV aerosol recovery and detection. Different sampling devices, parameters, and conditions were compared for optimal airborne virus sampling. Cutler et al. (2012) built upon this work by establishing a PRRSV aerosolization and treatment system with four channels (quartz tubes) for UVC disinfection. Another means of mitigating PRRSV was reported by La et al. (2019), where air ionization was applied on viral aerosols. Systems regarding aerosolization, inactivation, and collection of aerosols were also utilized in studies of other airborne viruses. Welch et al. (2018) established an aerosol exposure chamber (one-channel) where influenza virus was aerosolized, treated by UV, and then sampled by a biosampler; however, that system did not have engineering controls or regulation on airflow rate as well as pressure.

A large gap remains in the knowledge needed to develop effective and practical mitigation technologies for airborne pathogens. In particular, this research requires controlled systems under which mitigation technologies can be tested. We were motivated by the scarcity of research on aerosolized PRRSV and potential limitations of previous data collected without real-time monitoring of an aerosolization system. The objectives of this study were to:

(1) build and test a virus aerosolization system,
(2) provide a method and protocol of engineering control to regulate pressure and flow rate to ensure uniform aerosol generation and collection throughout all the experiments, as preparation for subsequent research on proper treatment (i.e., UV light) on aerosolized PRRSV,
(3) conduct mass balance calculations and uncertainty analyses to minimize variation and validate the system, and
(4) conduct PRRSV aerosolization and sampling experiments to verify the equivalency of the impingers.

This research aimed to provide a more quantitative, engineering perspective on controlling the process of aerosolization and collection of viral aerosols to better inform future research on treating aerosols and reduce uncertainty on the collected samples.

## Materials and Methods

### Experiment Setup (Obj. 1 and 2)

**FIGURE 1:**
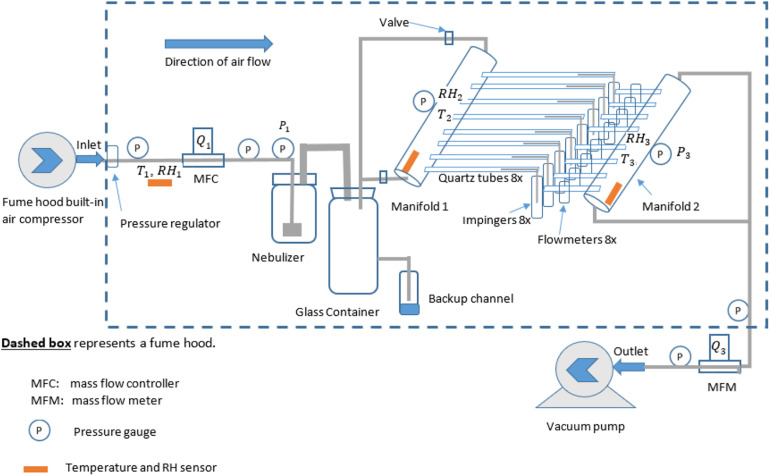
Experimental setup for generation and collection of airborne PRRSV inside a fume hood. The built-in air compressor in the fume hood was responsible for pressurizing air flowing into the system, and the right-side pump was vacuuming exhaust air coming out of the system. MFC, mass flow controller; MFM, mass flow meter.

The system (Figure 1 and Supplementary Figure 1) mainly consisted of three major sections: (i) aerosolization section, (ii) treatment section, and (iii) sampling section, following the direction of the airflow.

The (i) aerosolization section (Supplementary Figure 2) was responsible for generating PRRSV aerosols that were to be introduced to the treatment section. It included an air compressor (built-in with the fume hood), a pressure regulator, pressure gages, a mass flow controller (MFC) (model No.: GFCS-010013, Aalborg Instruments and Controls Inc., Orangeburg, New York, United States), a 24-jet collison nebulizer (formerly BGI Inc., now CH Technologies, Westwood, NJ, United States) (Supplementary Figure 3) with the addition of a pressure gage, and a glass container (12 L, or 3 gal) (Supplementary Figure 4). The pressure regulator included an air filter to remove dust before it came to MFC. The glass container has three channels, two of which were connected with manifolds (1 and 2), and the 3rd one was closed and considered emergency relief in case the inside pressure was too high for the system to handle (never used in the course of experiments).

The (ii) treatment section was designed to be used for subsequent aerosol treatment experiments (such as UV light, filtration, microwave). It included manifold 1 (with two pressure gages mounted on top) and all eight branches with quartz tubes and plastic connectors (see the construction of manifolds 1 and 2 in Supplementary Figures 5–8).

The (iii) sampling section was where the aerosols were collected in the sampling media [phosphate-buffered saline (PBS)]. It consisted of manifold 2 with eight identical branches connected to eight flowmeters (Catalog No. RMA-21-SSV, Dwyer Instruments Inc., Michigan City, IN, United States) (Supplementary Figures 9, 10), eight glass AGI 7541 impingers (Ace Glass Inc., Vineland, NJ, United States), pressure gages, a mass flow meter (MFM) (model No.: GFMS-010014, Aalborg Instruments and Controls Inc., Orangeburg, New York, United States), and a vacuum pump (VT 4.16 rotary vane vacuum pump, Becker Pumps Corp., Cuyahoga Falls, OH, United States) (Supplementary Figure 11). The AGI 7541 impingers have a designed flow rate of 6 L/min, and they were tested under experimental conditions where the actual flow rates were verified by Dwyer flowmeters.

All sections of the systems had sensors for temperature (*T*) and relative humidity (RH) (Table 1). Hose clamps reinforced all the connections between any fitting and plastic tubing to reduce the risk of leakage. According to the MFC and MFM manufacturer, the inlet air needed to be below 70% RH (met this requirement based on measurements) and with a particulate matter (PM) size of <50 (ensured by the filter in the pressure regulator). Both conditions were met in all trials.

**TABLE 1:** Summary of the monitoring sensors in the system and their specifications.

Parameters	Location	Sensor type	Label
Temperature and relative humidity	Ambient air	Omega smart temperature and humidity probe	*T_1_*, RH_1_
Temperature and relative humidity	Manifold 1	Omega smart temperature and humidity probe	*T_2_*, RH_2_
Temperature and relative humidity	Manifold 2	Omega smart temperature and humidity probe	*T_3_*, RH_3_
Pressure	Nebulizer	Analog pressure gage	*P_1_*
Pressure	Manifold 1	Omega digital pressure gage	*P_2_*
Pressure	Manifold 2	Omega digital pressure gage	*P_3_*
Airflow	System inlet	Aalborg mass flow controller (MFC)	*Q_1_*
Airflow	After impingers	Dwyer flowmeter	*Q*_st_
Airflow	System outlet	Aalborg mass flow meter (MFM)	*Q_3_*

Compressed air supplied by the fume hood passed through an MFC and then to a 24-jet collison nebulizer to pressurize the PRRSV inoculum and generate the aerosols. The nebulizer was prefilled with 60 ml of PRRSV inoculum, with 0.2% (*v*/*v*) antifoam A emulsion (Sigma-Aldrich Corp., St. Louis, MO, United States) known to reduce foaming and with no effect either on laboratory-cultured cells or PRRSV itself (Hermann et al., 2006). The nebulizer aerosolized 33∼37 ml of PRRSV inoculum in each (∼45-min) experiment.

Viral aerosols were then directed to the glass container and then into manifold 1, where they were distributed into eight branches (quartz tubes). The rear (right side) end of each quartz tube was connected to an impinger where the aerosols were captured. Each impinger was prefilled with 15 ml of PBS and 0.2% (*v*/*v*) antifoam A emulsion. Downstream of each impinger was a flow meter with an adjustable needle valve to ensure that all eight tubes had the same flow rates.

A vacuum pump (outside of the fume hood) forced constant airflow through manifold 2, confirmed by an MFM. During the experiment, the vacuum pump was turned on first, immediately followed by the compressed air valve. Once the reading of the sensors (MFC, pressure gage, etc.) became stable (within a few minutes), the timer for each experiment was started.

### A Theoretical Model for the Mass Balance of Airflow in Virus Aerosolization, Treatment, and Impingement System (Obj. 3)

The theoretical model assumes steady-state conservation of mass and energy during data collection. The purpose of this section is to present the % difference in the mass flow rate of dry air between inlet and outlet in order to detect significant air leakage in the system, to optimize the flow rate, to understand how an aerosolization system would work, and estimate the concentration of PRRSV in the sampling liquid.

#### Overall Mass Balance of Airflow Through the System

A mass balance for dry air is established for the aerosolization and impingement system,

(1) $${\dot{m}}_{a1}={\dot{m}}_{a3}$$

where ${\dot{m}}_{a1}$ is the mass flow rate of dry inlet air (kg_*a*_/s) and
${\dot{m}}_{a3}$is the mass flow rate of dry outlet air (kg_*a*_/s).

Mass flow rate is the product of the density times volumetric flow rate. Therefore, density and volumetric flow rates need to be determined.

(2) $${\dot{m}}_{a1}=\rho_{1}Q_{1}$$

(3) $${\dot{m}}_{a3}=\rho_{3}Q_{3}$$

where ρ_1_is the inlet air density (kg_*a*_/m^3^),
ρ_3_ is the outlet air density(kg_*a*_/m^3^),
*Q_1_* is the actual measured flow rate (m^3^/s) passing MFC,
and *Q_3_* is the actual measured flow rate (m^3^/s) passing MFM.

A general equation to correct the measured flow rate reading on the rotameters according to the pressure enacted on the flowmeter was used (Eq. 8).

#### Mass Balance of Water Vapor Through the Virus Nebulizer

**FIGURE 2:**
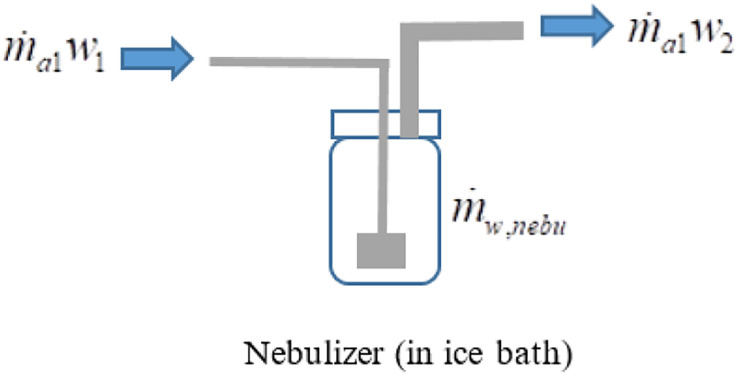
Mass balance of water vapor in the virus nebulizer. The inlet water vapor content plus the water vapor content generated in the nebulizer (from the virus inoculum) is equal to the water vapor content exiting the nebulizer.

The mass balance of moisture through the nebulizer (Figure 2) is:

(4) $${\dot{m}}_{a1}w_{1}+{\dot{m}}_{w,\text{nebu}}={\dot{m}}_{a1}w_{2}$$

where ${\dot{m}}_{w,\text{nebu}}$ is the change of water vapor content (kg_*w*_/s) caused by the nebulizer,
*w_1_* is the humidity ratio (kg_*w*_/kg_*a*_) of airflow before nebulizer (Figure 2), and
*w_2_* is the humidity ratio (kg_*w*_/kg_*a*_) of airflow after nebulizer (Figure 2)

Resulting in the theoretical water vapor content gained during the nebulization process,

(5) $${\dot{m}}_{w,\text{nebu}}={\dot{m}}_{a1}\left(w_{2}-w_{1}\right)$$

An online psychrometric calculator program (Marcks, 2006) was used to calculate the humidity ratio (*w*)and air density (ρ). Results are within the scope of ANSI/ASHRAE 41.6-1994 (ASHRAE, 2006). The input needed to yield these two variables (*w*,ρ)were the measured relative humidity (RH1, RH2, or RH3), standard temperature (defined as 21.1°C, based on instruction manuals from Aalborg and Dwyer), and standard pressure (defined as 14.7 psia, based on instruction manuals from Aalborg and Dwyer).

The air density (ρ) in Eqs. (2) and (3) is the air density at standard conditions (21.1°C and 14.7 psia), 1.19 kg_*a*_/m^3^. The variation of RH on ρ under standard conditions is negligible (<1%), and thus the change of ρ was ignored.

#### Mass Balance of Water Vapor Through the Impingers

**FIGURE 3:**
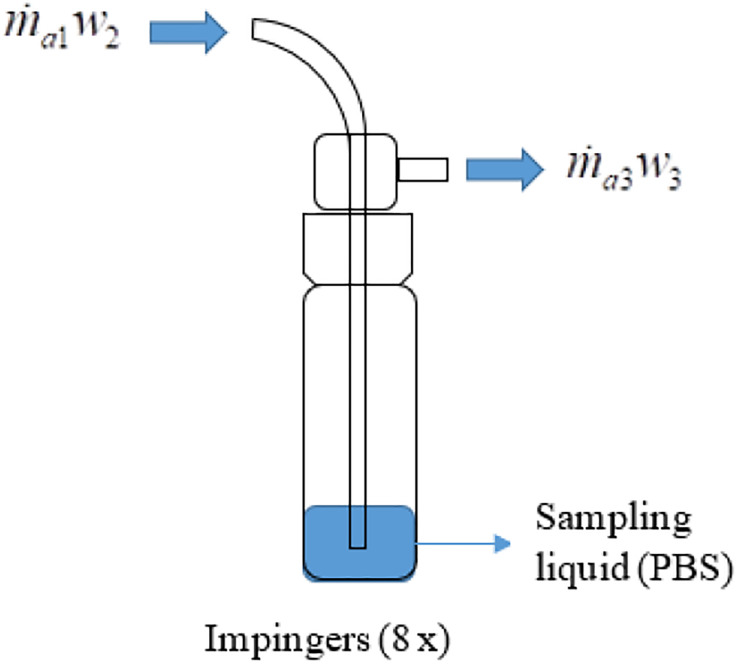
Mass balance of water vapor in the impinger to trap aerosolized virus into sampling liquid. A visual demonstration supplements Eq. (7); the water vapor content arriving in the impinger plus the water vapor content absorbed from the sampling liquid is equal to the water vapor content exiting the impinger.

The change of water vapor content across the eight impingers (Figure 3) can be expressed as,

(6) $${\dot{m}}_{a1}w_{2}+\sum_{n=1}^{8}{\dot{m}}_{w,\text{impg}}={\dot{m}}_{a3}w_{3}$$

where ${\dot{m}}_{w,\text{impg}}$ is the change of water vapor content (kg_*w*_/s) caused by each of the impingers.

After rearranging the terms,

(7) $$\sum_{n=1}^{8}{\dot{m}}_{w,\text{impg}}={\dot{m}}_{a3}w_{3}-{\dot{m}}_{a1}w_{2}$$

#### Mass Balance of Water Vapor Through the Flowmeters (Downstream From Impingers)

Airflow through each (*n* = 8) of the Dwyer flowmeters, corrected for actual *T* and *P* is:

(8) $$Q_{\text{st}}=Q_{\text{ob}}\sqrt{\frac{P_{a}\times T_{\text{st}}}{P_{\text{st}}\times T_{a}}}$$

where *Q*_st_ is the standard flow corrected for pressure and temperature,
*Q*_ob_ is the observed flowmeter reading (L/min),
*P_a_* is the measured absolute pressure: atmospheric pressure (14.7 psi) ± gage pressure (psi),
*P*_st_ is the standard pressure (14.7 psi),
*T_a_* is the measured temperature (Rankine scale, °R),
and *T*_st_ is the standard temperature (530°R).

Note that in Eq. (8), all the units under the square root sign are canceled by each other, so the only term that determines the units of this equation is *Q*_ob_. This is significant because Eq. (8) is also used in the mass balance and uncertainty analyses.

Introducing the constants in Eq. (8) results in,

(9) $$Q_{\text{st}}=Q_{\mathrm{ob}}\sqrt{\frac{\left(14.7+P_{a}\right)\times 530}{14.7\times \left(460+T_{a}\right)}}=Q_{\text{ob}}\sqrt{\frac{36.0544P_{a}+530}{T_{a}+460}}$$

Finally, combining the measurements from all eight flow meters yields,

(10) $$Q_{\text{tot}}=\sum_{n=1}^{8}Q_{\text{st}}$$

In the end, combining Eqs. (1), (5), and (7) results in the water vapor balance of each component in the system,

(11) $${\dot{m}}_{a1}w_{1}+{\dot{m}}_{w,\text{nebu}}+\sum_{n=1}^{8}{\dot{m}}_{w,\text{impg}}={\dot{m}}_{a3}w_{3}$$

(12) $${\dot{m}}_{a1}w_{1}+{\dot{m}}_{a1}\left(w_{2}-w_{1}\right)+\left({\dot{m}}_{a3}w_{3}-{\dot{m}}_{a1}w_{2}\right)={\dot{m}}_{a3}w_{3}$$

where ${\dot{m}}_{a1}w_{1}$ is the mass flow rate of dry air coming into the system multiplied by the humidity ratio of the air coming into the system and ${\dot{m}}_{a3}w_{3}$ is the mass flow rate of dry air coming out of the impingers multiplied by the humidity ratio of the air coming out of the impingers.

#### Stabilization of Aerosols: Calculation of Residence Time in the Glass Container

The aerosol residence time refers to the time that an aerosol particle stays in the glass container before it is directed to manifold 1. Assuming that the glass container is a “continuous stirred flow reactor” system (Cutler et al., 2013), the mass balance for the concentration of the target substance (e.g., aerosolized virus or tracer) (Table 2) in the container is:

**TABLE 2:** Steady-state conditions.

Time	$\frac{\text{C}}{{\text{C}}_{in}}$
1 τ (14 s)	1−*e*^−1^ = 63%
2 τ (28 s)	1−*e*^−2^ = 86%
3 τ (42 s)	1−*e*^−3^ = 95%
4 τ (56 s)	1−*e*^−4^ = 98%
5 τ (70 s)	1−*e*^−5^ = 99%

**The aerosol concentration with respect to the number of residence times in the glass container.**

(13) $$V\frac{\text{d}C}{\text{d}t}=QC_{\text{in}}-QC+\emptyset$$

where *V* is the volume of the glass container (L),
*C* is the concentration of the target aerosol in the container,
*C*_in_ is the concentration of the target aerosol entering the container,
*Q* is the volumetric flow rate (L/min),
and ∅ is the addition or subtraction of matters in the container in case of a reaction. In this model, it is assumed that there is no reaction in the container, so ∅ = 0.
*t* is the experiment or operation time (s)

Equation (13) can be rearranged to show the change of the aerosol concentration in the container,

(14) $$C=C_{\text{in}}\left(1-e^{-t\frac{Q}{V}}\right)$$

The aerosol residence time in the glass container, *τ* is,

(15) $$\tau =\frac{V}{Q}=\frac{11.4\text{L}}{48L/\min}=0.24\min=14s$$

**FIGURE 4:**
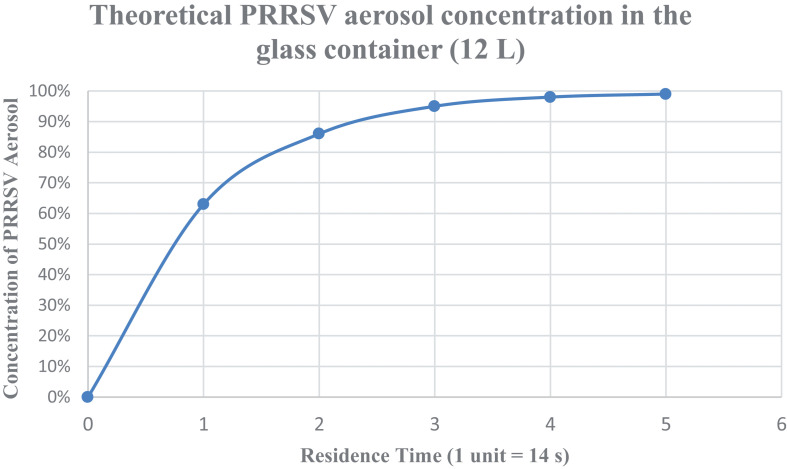
Steady-state conditions for the aerosolized virus. Theoretical PRRSV aerosol concentration in the glass container (12 L) with respect to the operation time (expressed in terms of the aerosol residence time within the glass container).

Based on Table 2 and Figure 4, the glass container was assumed to be at a steady state and contains 99% of the PRRSV aerosols, sufficient for stabilization purposes after 5 τ (70 s) of system operation. Therefore, after 70 s, the valves were turned on, and aerosols were directed into manifold 1.

### Uncertainty Analysis in the Mass Balance Model (Obj. 3)

First-order error analysis (FOEA), also known as the root sum of squares (RSS) method, was used to present the overall magnitude of measurement uncertainties. In the analysis below, θ refers to the sensitivity of the results, which was calculated by taking the partial derivative regarding each variable, while *U* refers to the individual measurement uncertainty (composed of both bias errors and precision errors). Adopted from Dettinger and Wilson (1981) and Zhang and Yu (2004), the overall uncertainty equation is established as follows,

(16) $$U_{\text{tot}}=\sqrt{\sum_{1}^{n}{\left(\theta_{n}U_{n}\right)}^{2}}$$

where *U*_tot_ is the total uncertainty of the equipment of study (e.g., a flowmeter),
θ_*n*_ is the sensitivity of the *n*th variable, which is calculated by taking the partial derivative of the equation (e.g., mass balance) with regard to variable *n*,
*U_n_* is the measurement uncertainty of the *n*th variable in the mass balance equation. This value is usually determined by the manufacturers of the measurement device.

#### Uncertainty Analysis of Mass Flow in the System Inlet and Outlet

For the overall system inlet and outlet, the mass flow rates are described in Eqs. (2) and (3). Since ρ_1_ and ρ_3_ were constants, the uncertainty was determined from *Q_1_* and *Q_3_*, which were measured by MFM and MFC, respectively. The uncertainty of either one is 1% of full scale reading, according to the manufacturers’ manual.

For MFC or MFM, the sensitivity and uncertainty of ${\dot{m}}_{a1}$ with regards to *Q_1_* are expressed as,

(17) $$\theta_{Q_{1}}=\frac{\partial {\dot{m}}_{a1}}{\partial Q_{1}}=\rho_{1}=1.19{\text{kg}}_{a}/{\text{m}}^{3}$$

(18) $$U_{Q_{1}}=accuracy\left(\%\right)\times F_{MFC}$$

where *F*_*MFC*_ is the full scale of the MFC (or MFM)

$$\left(U_{Q_{1}}=1\%\times 48L/\min=\pm 0.48L/\min=8\times 10^{-6}{\text{m}}^{3}/s\right)$$

Thus, the total uncertainty of MFC (for inlet), $U_{{\dot{m}}_{a1}}$ can be summarized as,

(19) $$U_{{\dot{m}}_{a1}}=\sqrt{{\left(\theta_{Q_{1}}U_{Q_{1}}\right)}^{2}}$$

The total uncertainty of MFM (for outlet) is the same as MFC because ρ_1_ = ρ_3_ = 1.19kg_*a*_/m^3^ (based on the assumption made in section “Mass Balance of Water Vapor Through the Virus Nebulizer”), *U*_*Q*1_ = *U*_*Q*3_ = 4.8 × 10^−4^ m^3^/s; therefore, the calculation is the same as above. In this case, the uncertainty for mass flow inlet and outlet is constant, which does not change due to environmental conditions. Based on the known values and taking them into Eq. (19), results in $U_{{\dot{m}}_{a1}}=U_{{\dot{m}}_{a3}}$ = 9.52×10^−6^ kg_*a*_/s.

#### Uncertainty Analysis of Mass Flow Through Each Flowmeter (Quartz Tube)

The flowrate of each Dwyer flowmeter is expressed as follows (derived from Eq. 8),

(20) $$Q_{\text{st}}=Q_{\text{ob}}\sqrt{\frac{\left(14.7+P_{a}\right)\times 530}{14.7\times \left(460+T_{a}\right)}}=Q_{\text{ob}}\sqrt{\frac{36.0544P_{a}+530}{T_{a}+460}}$$

The mass flow (${\dot{m}}_{a2}$) through each quartz tube is,

(21) $${\dot{m}}_{a2}=\rho_{2}Q_{\text{ob}}\sqrt{\frac{36.0544P_{3}+530}{T_{3}+460}}\;$$

For each of the variables in Eq. (21), its θ_*n*_ (the sensitivity) is calculated by taking the partial derivative of Eq. (21) with respect to that variable, *n*,

(22) $$\theta_{Q_{\text{ob}}}=\frac{\partial {\dot{m}}_{a2}}{\partial Q_{\text{ob}}}=\rho_{2}\sqrt{\frac{36.0544P_{3}+530}{T_{3}+460}}$$

(23) $$U_{Q_{\text{ob}}}=\text{accuracy}\left(\%\right)\times FS_{f}$$

where FS*_*f*_* is the full scale of the flowmeter (RMA-21-SSV), and thus *U*_*Q*ob_ = ± 3% × 10L/*min* = ± 0.3L/*min* = 5 × 10^−6^ m^3^/s.

(24) $$\theta_{P_{3}}=\frac{\partial {\dot{m}}_{a3}}{\partial P_{3}}=\frac{18.0272Q_{\text{ob}}\rho_{2}}{\sqrt{\left(36.0544P_{3}+530\right)\left(T_{3}+460\right)}}$$

(25) $$U_{P_{3}}=\text{accuracy}\left(\%\right)\times FS_{p}$$

where FS*_*p*_* is the full scale of the pressure gage, and thus *U*_*P*3_ = ± 2% × 30 psi = ± 0.6 psi

(26) $$\theta_{T_{3}}=\frac{\partial Q_{\text{st}}}{\partial T_{3}}=\frac{\left(-18.0272P_{3}-265\right)Q_{\text{ob}}\rho_{2}}{{\left(T_{3}+460\right)}^{2}\sqrt{\frac{36.0544P_{3}+530}{T_{3}+460}}}$$

(27) $$\theta_{\rho_{2}}=\frac{\partial Q_{\text{st}}}{\partial \rho_{2}}=Q_{\text{ob}}\sqrt{\frac{36.0544P_{3}+530}{T_{3}+460}}$$

In addition, *U*_*T*3_ = ± 0.2°C of reading, *U*_ρ2_ = ± 2.25% of reading.

Thus, the total uncertainty for ${\dot{m}}_{a2}$, the mass flow through each quartz tube (measured Dwyer flowmeters) is expressed as,

(28) $$U_{{\dot{m}}_{a2}}=\sqrt{{\left(\theta_{Q_{\mathrm{ob},}}U_{Q_{\text{ob}}}\right)}^{2}+{\left(\theta_{P_{3}}U_{P_{3}}\right)}^{2}+{\left(\theta_{T_{3}}U_{T_{3}}\right)}^{2}+{\left(\theta_{\rho_{2}}U_{\rho_{2}}\right)}^{2}}$$

### Example Calculation of the Uncertainty Analysis (Obj. 3)

The summary of measured values for the mass balance model and uncertainty analysis are shown in Table 3.

**TABLE 3:** Sample data collection for all variables needed for mass balance and uncertainty analysis.

Location	Variable	Reading	Uncertainty	Measurement device
Inlet/ambient	*T_1_*, RH_1_	21.0°C, 15.9%	±0.3°C of reading, ±2% of reading	Omega smart temperature and humidity probe
System inlet	*Q_1_*	47.9 L/min	±1% FS	Aalborg mass flow controller
Nebulizer	*P_1_*	21.5 psi	±0.25% FS	Pressure gage
Manifold 1	*P_2_*	0 psi	2% FS for the middle half	Pressure gage
Manifold 1	*T_2_*, RH_2_	20.2°C, 84.6%	±0.3°C of reading, ±2% of reading	Omega smart temperature and humidity probe
Manifold 2	*P_3_*	−4.85 psi	2% FS for the middle half	Vacuum gage
Manifold 2	*T_3_*, RH_3_	20.8°C, 16.7%	±0.3°C of reading, ±2% of reading	Omega smart temperature and humidity probe
Postimpinger	*Q*_ob_	7.25 L/min*	±3% of reading	Dwyer flowmeter
System outlet	*Q_3_*	49.0 L/min	±1% FS	Aalborg mass flow meter
Ice buckets**	–	0∼2°C	N/A	Thermocouples

*The actual flowrate (Q_*st*_*) was derived from the observed flow rate (Q*_*ob*_*) using Eq. (8). In this case*,*Q*_*st*_was 5.9 L/min. **Ice bath (container with water and ice mixture) was provided for nebulizer and impingers so that their temperature can be maintained around 0°C to extend the survivability of PRRSV. T_*n*_, temperature (°C); RH_*n*_, relative humidity (%); P_*n*_, pressure (Pa); Q_*n*_, flow rate (L/min); FS, full scale.

Taking the values from Table 3 above into Eqs. (4) and (5) results in m.a1 9.50×10-4kga/s, ${\dot{m}}_{a3}=9.72\times 10^{-4}{\text{kg}}_{a}/\text{s}$. Therefore, the percentage difference of mass flow between the entire system inlet and outlet was, $\frac{{\dot{m}}_{a3}-{\dot{m}}_{a1}}{{\dot{m}}_{a1}}\times 100\%$ = 2.32%. This indicates relatively low variability and a good mass balance closure. This illustrates sufficient quality control in the assembled and tested virus aerosolization system.

For each quartz connected to a Dwyer flowmeter, based on Eq. (28), the uncertainty is $U_{{\dot{m}}_{a2}}$ = 7.72 × 10^−6^ kg_*a*_/s. According to Eq. (21), ${\dot{m}}_{a2}$ = 1.18 × 10^−4^ ± 7.72 × 10^−6^ kg_*a*_/s, or 1.18 × 10^−4^ kg_*a*_/s ± 6.54%. Thus, the uncertainty is minimal, and it does not affect the accuracy of engineering control.

### Confirmation of Uniform Airflow Distribution Among Treatments (Obj. 2 and 4)

Rhodamine B (Sigma-Aldrich Corp., St. Louis, MO, United States), a fluorescent physical tracer (excitation wavelength = 544 nm, emission wavelength = 576 nm) that is not harmful to mammalian cells and viruses (Hermann et al., 2006), was used to confirm the uniformity of the airflow across the eight tubes before the experiments on PRRSV began. Rhodamine B was dissolved in PBS to achieve a concentration of 2 ppm and 60 ml, placed into a 24-jet collison nebulizer and aerosolized in each experiment. Before each experiment, the system was tested with PBS (no rhodamine B added), and the Dwyer flowmeters were adjusted to ensure that their readings were the same. After the 10-min experiment, 2 ml of liquid from each impinger was transferred into polystyrene cuvettes (Model No. 2405, Stockwell Scientific, Scottsdale, AZ, United States) and warmed up for 10 min in the dark so that they rose to room temperature. The fluorescent intensity was measured by a Modulus Fluorometer (Model 9200-000, Turner Biosystems Inc., Sunnyvale, CA, United States) which was equipped with a green optical kit (Model 9200-042, Turner BioSystems Inc., Sunnyvale, CA, United States). Each sample was distributed into three aliquots (2 ml each), with three measurements each, and the average value was recorded.

### PRRSV Propagation, Isolation, and Virus Titer Determination (Obj. 4)

The PRRSV used in this experiment (MN-184, PRRSV-2 Lineage 1) was provided by the Veterinary Diagnostic Laboratory (College of Veterinary Medicine, Iowa State University). The virus was propagated in the MARC-145 cell line, a clone of the African monkey kidney cell line MA-104 (Kim et al., 1993). MARC-145 cells were cultured in the RPMI-1640 medium supplemented with 10% fetal bovine serum, 2 mM L-glutamine, 0.05 mg/ml gentamicin, 100 unit/ml penicillin, 100 μg/ml streptomycin, and 0.25 μg/ml amphotericin. Large-scale virus propagation was conducted in five-layer flasks (Thermo Fisher Scientific, Rochester, NY, United States). Briefly, when cells reached 80–90% confluence, stock PRRSV was added. The estimated number of cells at that range of confluency was 18.6–21.0 × 10^6^ cells/layer (Thermo Fisher Scientific – US, 2021). Flasks were observed daily for cytopathic effect (CPE). When the CPE was abundant (5–7 days), flasks underwent two freeze-thaw cycles, followed by centrifugation at 4,200×*g* for 10 min to harvest the supernatant. Approximately 3 L of virus inoculum was harvested with a geometric mean virus titer of 10^5.56^ TCID_50_/ml. The inoculum was thoroughly mixed to ensure homogeneity, distributed into 30 ml aliquots and stored at −80°C.

For determination of TCID_50_ in research samples, impinger fluid was transferred to a biosafety cabinet in a BSL-2 laboratory, and tenfold serial dilutions were performed, with eight replicates for each sample. Each well (in 96-well plates) was prefilled with 270 μl of RPMI-1640 medium, and then the sample was added to the first row of the plates; the solution was mixed, and then 30 μl of liquid transferred sequentially from one row to another. Thus, the dilution for each row ranged from 10^0^, 10^–1^, …, to 10^–7^, respectively. We considered the 10^0^ dilutions to improve method detection limits for the benefit of the subsequent experiments on PPRSV survival after UV treatment. Thereafter, 100 μl from each well was inoculated into 80∼90% subconfluent MARC-145 cells grown in 96-well plates. The plates were incubated at 37°C in a humidified 5% CO_2_ incubator. CPE development was checked under a microscope daily, and infected wells were marked as positive until no more additional wells were identified as infected (5–7 days). To confirm the presence of PRRSV, the plates were fixed (80% acetone for 10 min), dried, and stained with a PRRSV nucleocapsid protein-specific monoclonal antibody (SDOW17-F) conjugated to fluorescent isothiocyanate (Rural Technologies, Inc., Brookings, SD) for 1 h at 37°C. The antibody conjugate was decanted, and the cell plates were washed with PBS (1× pH 7.4) three times, 5 min each time. Plates were read under an Olympus IX71 fluorescent microscope (Olympus America Inc., Center Valley, PA, United States). The Spearman-Karber method (Karber, 1931; Lei et al., 2020) was used to calculate the virus titers, which were based on the number of wells showing positive PRRSV-specific fluorescence at specific dilution and the results expressed as TCID_50_ per milliliter of the impinger fluid (Cutler et al., 2012; Yim-Im et al., 2021).

### Statistical Analysis

The statistical analysis was conducted using JMP Pro (Version 15, SAS Institute Inc., Cary, NC, United States). One-way ANOVA and Tukey’s test was used to determine statistical significance. *p*-values regarding the virus titer were calculated and compared between each combination of two trials (out of the total of three trials). If a *p*-value was <0.05, the results were considered significantly different.

## Results

### Verification of Engineering Control on Airflow Rate in the System (Obj. 3)

During each experiment, environmental data such as temperature, pressure, and flow rate were measured and recorded. The results were summarized in Table 4. Table 4 summarized the average values (with ± standard deviation) of all of the environmental data of the (*n* = 3) three trials with PRRSV. The purpose of Table 4 is to show the level of engineering control and environmental conditions that the system had, and the variation of the data was indeed minimal. The inlet airflow rate was controlled and measured by the MFC. The inlet air pressure and pressure in the nebulizer were maintained at ∼20 psi (1.4 atm).

**TABLE 4:** Environmental data for all experimental trials on aerosolization and collection of PRRSV.

Environmental parameters (mean ± St. Dev.)			
Engineering-controlled parameters	Inlet air flow rate (L/min), *Q_1_*	47.9 ± 0.4	
	Inlet air pressure (psi), *P_1_*	24.6 ± 0.6	
	The air pressure in the nebulizer (psi), *P_2_*	20.7 ± 0.6	
	The pressure at Manifold 2 (psi), *P_3_*	−4.9 ± 0.4	
	Outlet airflow rate (L/min), *Q_3_*	49.1 ± 0.1	
	Total airflow (L)	2,157.0 ± 16.6	
	Average airflow rate per treatment (L/min)	5.8 ± 0.1	
	Total airflow per treatment (L)	262.5 ± 4.5	
Monitored parameters (uncontrolled)	Ambient air: temperature (°C) and RH (%),*T_1_*, RH_1_	21.1 ± 0.7	41.6% ± 6.4%
	Manifold 1 temperature (°C) and RH (%),*T_2_*, RH_2_	20.1 ± 0.5	80.0% ± 2.2%
	Manifold 2 temperature (°C) and RH (%),*T*_3_, RH_3_	20.6 ± 0.2	36.3% ± 3.8%

**The system was designed to control and minimize variations of physical parameters. St. Dev., standard deviation.**

Table 5 presents TCID_50_ estimates of PRRSV concentration in the nebulizer and impingers. The comparison showed that a fraction of the PRRSV present in the nebulizer was subsequently recovered in the impingers. This reflects the inactivation of the infectious virus during the process of nebulization, in the aerosolized state, by the process of impingement, and during sample handling, as well as the inefficiency of impingement itself.

**TABLE 5:** Measured and estimated TCID_50_ values from the medium in the nebulizer and in impingers.

	Nebulizer	Impinger (8×)	Ratio (nebulizer/impinger)
PRRSV titer* (log_10_(TCID_50_)/ml)	5.563	3.798**	58:1
Estimated No. of TCID_50_ aerosolized or recovered (log_10_(TCID_50_))	7.107	5.877	17:1
Description	35 ml of virus inoculum aerosolized for 45 min per experiment	15 ml of medium per impinger (8× in total per experiment)	–

*Estimated as the geometric mean for nebulizer and impingers. **The geometric mean of PRRSV titer in the impinger fluid from the (n = 3) trials.

Rhodamine B was used as a tracer (positive control) to account for variation among treatments (Table 6). All trials had relative standard deviations (RSD) <12%. These three trials were independent of the trials mentioned in Table 3, and the only purpose was to evaluate the system integrity before aerosolizing PRRSV. The RSD below 12% for the Rhodamine dye is acceptable in the context of virology research. Inherent variability is assumed for work on biological systems that is generally higher than in engineering fields.

**TABLE 6:** Pre-PRRSV experimental verification of air flowrate in each treatment (quartz tubes 1–8) with rhodamine B.

	Prep trial 1		Prep trial 2		Prep trial 3	
Sample source	Fluorescent intensity (FSU)	Percentage difference (%)?**	Fluorescent intensity (FSU)	Percentage difference (%)?**	Fluorescent intensity (FSU)	Percentage difference (%)?**
Treatment 1	5.11×10^3^	−1.3%	5.41×10^3^	14.7%	4.09×10^3^	0.7%
Treatment 2	4.73×10^3^	−8.6%	4.63×10^3^	−2.0%	4.91×10^3^	20.9%
Treatment 3	4.56×10^3^	−12.0%	4.01×10^3^	−15.1%	3.56×10^3^	−12.5%
Treatment 4	5.70×10^3^	10.1%	4.84×10^3^	2.6%	3.75×10^3^	−7.8%
Treatment 5	5.28×10^3^	2.0%	5.02×10^3^	6.3%	3.48×10^3^	−14.4%
Treatment 6	5.08×10^3^	−1.9%	4.50×10^3^	−4.7%	4.17×10^3^	2.6%
Treatment 7	5.32×10^3^	2.8%	4.86×10^3^	3.0%	4.53×10^3^	11.4%
Treatment 8	5.64×10^3^	8.9%	4.49×10^3^	−4.9%	4.03×10^3^	−0.9%
Mean	5.18×10^3^	–	4.06×10^3^	–	4.06×10^3^	–
St. Dev.	3.99×10^2^	–	4.54×10^2^	–	4.54×10^2^	–
RSD	7.7%	–	8.3%	–	11.2%	–

FSU, fluorescent standard unit; St. Dev., standard deviation; RSD, relative standard deviation. **Percentage difference (%): deviated from the average value of the eight samples (of treatments).

### Verification of the Uniformity of Airborne PRRSV Titer Across the Treatments (Obj. 3)

The virus titer of the impinger fluid collected from each trial was expressed in the form of log_10_ normalization (Table 7). Mean, geometric mean, and standard deviations were calculated to evaluate the variability within and between the experimental trials. Data in Table 7 was used to estimate the total geometric mean of PRRSV titer in impingers (i.e., “3.798” reported in Table 5).

**TABLE 7:** Experimental verification of uniformity of the PRRSV titer (log_10_(TCID_50_)/ml) in the sampling fluid among identical eight treatments.

	PRRSV titer(log_10_(TCID_50_)/ml)		
Sample source	Trial 1	Trial 2	Trial 3
Treatment 1	3.88	3.88	4.00
Treatment 2	3.88	4.13	3.38
Treatment 3	4.13	4.00	3.25
Treatment 4	3.88	3.63	3.63
Treatment 5	3.75	4.13	4.13
Treatment 6	3.88	4.00	3.13
Treatment 7	3.75	3.88	3.63
Treatment 8	3.88	3.63	3.75
Mean ± St. Dev.	3.88 ± 0.11	3.91 ± 0.18	3.61 ± 0.33
Geo mean ± St. Dev.	3.88 ± 0.11	3.90 ± 0.19	3.59 ± 0.33
RSD	2.8%	4.7%	9.1%
Least square mean	3.88	3.91	3.61
SD error	0.085		
Lower 95%	3.70	3.73	3.44
Upper 95%	4.06	4.09	3.79

**St. Dev., standard deviation; Geo Mean, geometric mean; RSD, relative standard deviation (based on mean and st. dev.); SD error, standard error.**

Statistical analysis on virus titer (both numerical and log_10_-based values) was summarized in Table 8.

**TABLE 8:** Tukey’s test on the statistical significance of the three trials.

	*p*-value for virus titer (TCID_50_/ml)	*p*-value for virus titer (log_10_(TCID_50_)/ml)
Trial 2 vs. trial 3	0.1479	0.0560
Trial 1 vs. trial 3	0.3572	0.0934
Trial 2 vs. trial 1	0.8458	0.9638

**p-values were shown for the three comparisons.**

**FIGURE 5:**
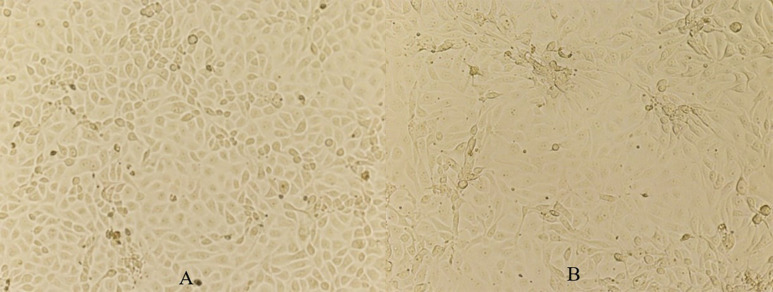
Views of MARC-145 cells at 3 days postinoculation with control impinger fluid **(A)** and with the collected PRRSV impinger fluid **(B)**. Magnification, ×160.

**FIGURE 6:**
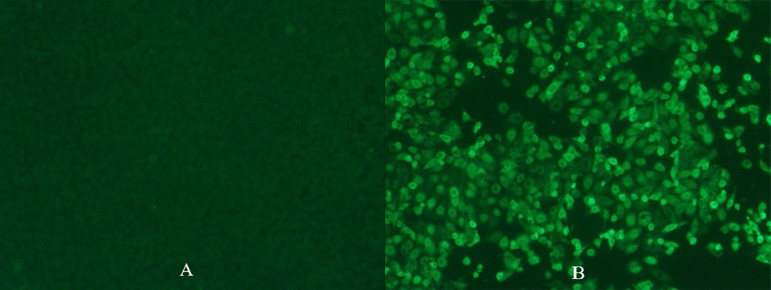
Immunofluorescence staining of cells inoculated with control impinger fluid **(A)** or PRRSV impinger fluid **(B)** with PRRSV-specific antibody conjugated to FITC. Pictures were taken on 6 days postinoculation when the MARC-145 cell plates were fixed using 80% acetone and stained by SDOW-17 PRRSV FITC conjugate. Magnification, ×160.

Figure 5 shows the morphological changes of MARC-145 cells under an optical microscope on 3 days postinoculation of the cells with the sample. In Figure 5A where cells were inoculated with the control impinge fluid, i.e., virus-negative PBS, no CPE was observed. In contrast, in Figure 5B where cells were inoculated with the collected PRRSV impinger fluid, CPE characterized by enlargement and clumping of cells was observed. The cell plates were then placed back into the incubator for continuous CPE development until 5–7 days postinoculation. At that time, cells were fixed and stained with FITC-conjugated PRRSV-specific antibody to confirm the infection status of the cells. Examples of immunofluorescence staining are shown in Figure 6. No immunofluorescence staining was observed in the cells inoculated with the control impinger fluid (Figure 6A). In contrast, in the cells inoculated with the collected PRRSV impinger fluid, positive staining by PRRSV-specific antibody conjugate was clearly observed (Figure 6B).

## Discussion

### Sources of Variation

Sources of variation in the data included engineering factors, e.g., sensor measurements (temperature, relative humidity, flow rate), physical tracer (Rhodamine B), and biological factors, e.g., biologically based estimates of virus titer. In the engineering aspect, an uncertainty analysis was conducted with respect to sensor measurements, and the goal was to control and minimize the uncertainty. Ultimately, low variation in the engineering controls minimizes the risks of unacceptable biological variability (which is inherently higher).

### Reproducibility

The Rhodamine B fluorometric data supported the conclusion that the airflow was uniform among the eight treatments (tubes) (Table 6), i.e., the RSD among all eight treatments (tubes) was <12%, given all the engineering controls mentioned in the previous sections. However, the absolute values of the fluorescence intensity varied among different trials and cannot be used as verification of flow rate because the variation of flow rate (controlled and verified by MFC, MFM, Dwyer flowmeters) was almost negligible between any two trials (section “Example Calculation of the Uncertainty Analysis (Obj. 3)”).

The PRRSV TCID_50_ estimation of infectious virus is a complex quantal assay that requires (*a*) the propagation of cell cultures, (*b*) inoculation of cells with the virus, and (*c*) visual assessment of the infection of cells by the virus. Despite the biological complexities, TCID_50_ estimates were relatively consistent within and between trials (Table 7).

The system provided acceptable control of flow rate, pressure, and other physical factors while minimizing the potential effect on the microinfectivity assay. The follow-up research on the inactivation of aerosolized PRRSV using UV-C and UV-A irradiation is described by Li et al. (2021). The same type of studies (aerosolization and recovery) and mitigation could be done using other important airborne pathogens.

### Limitations

The system can only control with a flow rate that is <50 L/min; more specifically, the inlet air flow rate was controlled to be around 48 ± 0.5 L/min. For a higher flow rate, this system would not be practical to handle and would require a redesign, testing, and commissioning similar to the approach presented herein.

The selection of impingers was based on the flow rate, reliability, and cost. Multiple models of impingers were considered, such as SKC BioSampler, AGI 7541, AGI 7542 (also known as AGI 30), etc. Eventually, AGI 7541 was chosen because its designed flow rate is 6 L/min, only half of the designed flow rate of SKC BioSampler or AGI 7542. The lower flow rate impingers allowed for greater flexibility in evaluating UV light’s effect on infectious PRRSV in the subsequent research (Koziel et al., 2020; Li et al., 2021). On the one hand, a lower flow rate reduces the efficiency of sampling aerosols. However, on the other hand, a lower flow rate allows more treatment time for the aerosols that are suspended in the air before being captured by the impingers. Thus, the subsequent study (i.e., testing the UV light to inactivate the PRRSV aerosol) benefitted from a wider range of treatment times available (Koziel et al., 2020; Li et al., 2021).

The majority cost of the system was attributed to the sensors, especially the Aalborg MFC and MFM. The Omega Smart Probes were the second most expensive, while the electronic pressure gages were the third. Some cost savings could be realized by using less expensive sensors (as described in Supplementary Material 1). However, we highly recommend conducting the error propagation and uncertainty analyses as part of the decision-making process.

Overall, the presented system construction and protocol for aerosolization and sampling of PRRSV could be applicable to the aerosolization of other viruses. This experiment could be helpful for future research on virus aerosolization, treatment, and sampling in both human and animal health.

## Conclusion

A system for virus aerosolization and protocol for engineering control to regulate pressure and flow rate to ensure uniform aerosol generation and collection was described. The system for virus aerosolization was built and tested (Obj. 1). The uniform airflow distribution was confirmed by the physical tracer (<12% RSD) for all the treatments and sound engineering control of flow rates (Obj. 2). Theoretical uncertainty analysis and mass balance calculation to identify potential variations and reduce them were conducted to prove that a <3% loss of air mass flow rate between the inlet and outlet (Obj. 3). The results showed that the PRRSV titers collected from the impingers were not statistically different, indicating that the variations were acceptable. This indicated the readiness of the system for research on virus aerosolization and treatment (e.g., by ultraviolet light). The TCID_50_ values of the collected impinger fluids showed no statistical difference between any two of the three trials (*p*-value = 0.148, 0.357, 0.846) (Obj. 4). The presented virus aerosolization system and protocol are also potentially useful for research of other types of airborne pathogens and their mitigation on a laboratory scale.

## Data Availability Statement

The original contributions presented in the study are included in the article/Supplementary Material, further inquiries can be directed to the corresponding author/s.

## Author Contributions

JK, JJZ, SH, and WJ: conceptualization and funding acquisition. PL, T-YC, and BC: formal analysis. PL, T-YC, and ML: investigation. PL, JK, JJZ, JZ, WY-I, and T-YC: methodology. JK, JJZ, and JZ: resources and supervision. PL, JK, JJZ, SH, ML, JZ, and WJ: validation. PL: visualization and writing (original draft). PL, JK, JJZ, SH, JZ, and WJ: writing (review and editing). All authors contributed to the article and approved the submitted version.

## Conflict of Interest

The authors declare that the research was conducted in the absence of any commercial or financial relationships that could be construed as a potential conflict of interest.

## Supplementary Material

The Supplementary Material for this article can be found online at: https://www.frontiersin.org/articles/10.3389/fbioe.2021.659609/full#supplementary-material. The Supplementary Material is organized as follows: uncertainty analyses for a low-cost alternative system for virus aerosolization (section 1.1), followed by Supplementary Figures 1–11 illustrating details of the aerosolization system and its construction.
