# Supplementary material for: Designing and Testing of a System for Aerosolization and Recovery of Viable Porcine Reproductive and Respiratory Syndrome Virus (PRRSV): Theoretical and Engineering Considerations
Source: Front Bioeng Biotechnol. 2021 May 10;9:659609. doi: 10.3389/fbioe.2021.659609 (PMC8141751; doi:10.3389/fbioe.2021.659609)
Supplement: Supplementary file 1 [file Data_Sheet_1.pdf]

Supplementary Material

1 Uncertainty analyses for a low-cost alternative system for virus aerosolization

Before the implementation of the current system mentioned in the Materials and Methods section, an initial system was constructed similarly except that the Aalborg mass flow meter (MFM) and mass flow controller (MFC) were replaced by Dwyer flowmeters. Below is the uncertainty analysis of this type of low-cost system. Since MFM and MFC have higher prices than mechanical flowmeters, this could be an alternative way of commissioning the experiment.

Adapted from section 2.2 (main manuscript), the inlet mass flow rate entering the system can be expressed as,

$$\dot{m}_{a1} = \frac{Q_{ob,1}}{v_1}\sqrt{\frac{36.0544P_1 + 530}{T_1 + 460}} \quad [29]$$

the sensitivity and uncertainty of each of the variables can be expressed as,

θ is calculated by taking the partial derivative regarding each variable,

$$\theta_{Q_{ob,1}} = \frac{\partial \dot{m}_{a1}}{\partial Q_{ob,1}} = \frac{1}{v_1}\sqrt{\frac{36.0544P_1 + 530}{T_1 + 460}} \quad [30]$$

$$U_{Q_{ob,1}} = \pm\, 3\% \times 40\ L/\min = \pm\, 1.2\ L/min = 2 \times 10^{-5}\ m^3/s \quad [31]$$

(Accuracy × Full Scale of the Dwyer flowmeter model VFB-69-BV)

$$\theta_{P_1} = \frac{\partial \dot{m}_{a1}}{\partial P_1} = \frac{18.0272Q_{ob,1}}{v_1\sqrt{(36.0544P_1 + 530)(T_1 + 460)}} \quad [32]$$

$$U_{P_1} = \pm\, 2\% \times 30\ psi = \pm\, 0.6\ psi \quad [33]$$

(Accuracy × Full Scale of the pressure gauge)

$$\theta_{T_1} = \frac{\partial Q_1}{\partial T_1} = \frac{(-18.0272P_1 - 265)Q_{ob,1}}{(T_1 + 460)^2 v_1\sqrt{\frac{36.0544P_1 + 530}{T_1 + 460}}} \quad [34]$$

$$U_{T_1} = \pm\, 0.2\ ℃\ \text{of reading}$$

$$\theta_{v_1} = \frac{\partial Q_1}{\partial v_1} = -\frac{Q_{ob,1}\sqrt{\frac{36.0544P_1 + 530}{T_1 + 460}}}{{v_1}^2} \quad [35]$$

$$U_{v_1} = \pm\, 2.25\%$$

$$U_{\dot{m}_{a1}} = \sqrt{\left(\theta_{Q_{ob,1}} U_{Q_{ob,1}}\right)^2 + \left(\theta_{P_1} U_{P_1}\right)^2 + (\theta_{T_1} U_{T_1})^2 + (\theta_{v_1} U_{v_1})^2} \quad [36]$$

Engineering considerations: PRRSV aerosolization & collection

Supplementary Table 1 shows the measured variables used for a sample calculation.

Supplementary Table 1. Mass flow model verification based on measured variables.

Variables	Reading	Uncertainty	Position	Measurement Device
T_1, RH_1	24.1 °C, 25.8%	± 0.2 °C of reading, ± 2% of reading	Inlet air (downstream from the pump)	Govee T & R.H. meter
P_1	14.7+6.5 psi	2% F.S. for the middle half	Inlet air (downstream from the pump)	Pressure gauge
Q_1	28 LPM (before correction)	± 3% FS	System inlet	Dwyer flow meter (4 – 40 LPM)
P_2	0 psi (normal)	2% F.S. for the middle half	Manifold 1	Pressure gauge
T_2, RH_2	26.7 °C, 57.2%	± 0.2 °C of reading, ± 2% FS	Manifold 1	Govee T & RH meter
Single tube flow rate	6.5 LPM LPM (after correction)	± 3% FS	Manifold 2	Dwyer flow meter (0 – 10 LPM)
P_3	-10 inHg (-5 psi)	2% FS for the middle half	Manifold 2	Vacuum gauge
T_3, RH_3	26.1 °C, 54.2%	± 0.2 °C of reading, ± 2% FS	Manifold 2	Govee T & RH meter
Ice bucket temperature	0 ~ 2 °C	N/A	Ice buckets	Thermocouples
Q_3	40 LPM (before correction)	± 3% FS	System outlet	Dwyer flow meter (0 – 100 LPM)

$$Q_1 = Q_{ob,1}\sqrt{\frac{36.0544P_1+530}{T_1+460}} \quad [37]$$

Plugging in $Q_{ob,1}$ = 28 L/min, P_1 = 6.5 psi, T_1 = 75 °F to Eqn. [37], Q_1 = 33.5 L/min,

Plugging in standard temperature and standard pressure, and RH_1= 25.8% to the psychometric calculator, the result is that v_1= 0.839 m^3/kg,

$$\dot{m}_{a1} = \frac{Q_1}{v_1} = 6.65 \times 10^{-4}\ kg_a/s \quad [38]$$

$$Q_3 = Q_{ob,3}\sqrt{\frac{36.0544P_3+530}{T_3+460}} \quad [39]$$

Plugging in $Q_{ob,3}$ = 40 L/min, P_3 = -5 psi, T_3 = 79 °F to Eqn. [39], Q_3 = 32.2 LPM,

Plugging in standard temperature and standard pressure, and RH_3= 54.2% to the psychometric calculator, the result is that v_3= 0.845 m^3/kg,

$$\dot{m}_{a3} = \frac{Q_3}{v_3} = 6.35 \times 10^{-4}\ kg_a/s \quad [40]$$

$$\dot{m}_{a3} = \frac{Q_{ob,3}}{v_3}\sqrt{\frac{36.0544P_3+530}{T_3+460}} \quad [41]$$

$$\%\ difference = \frac{\dot{m}_{a1}-\dot{m}_{a3}}{\dot{m}_{a1}} \times 100\% = 4.51\% \quad [42]$$

So, there is 4.51% loss of mass flow rate between $\dot{m}_{a1}$ and $\dot{m}_{a3}$.

$$\dot{m}_{w,nebu} = \dot{m}_{a1}(w_2 - w_1) \quad [43]$$

w_1 = 0.00331 kg_w/kg_a

w_2 = 0.0107 kg_w/kg_a

And thus $\dot{m}_{w,nebu} = 3.27 \times 10^{-5}\ kg_w/s$

$$\dot{m}_{w,impg} = \dot{m}_{a3}w_3 - \dot{m}_{a1}w_2 \quad [44]$$

w_3 = 0.01753 kg_w/kg_a

And thus $\dot{m}_{w,impg} = -4.02 \times 10^{-6}\ kg_w/s$

Change of water vapor content in the system,

$$\dot{m}_{w,nebu} + \dot{m}_{w,impg} = 4.22 \times 10^{-6}\ kg_w/s \quad [45]$$

Propagation of error (plugging in numbers):

$$\theta_{Q_{ob,1}} = \frac{\partial \dot{m}_{a1}}{\partial Q_{ob,1}} = \frac{1}{v_1}\sqrt{\frac{36.0544P_1+530}{T_1+460}} = 2.04\ kg_a/m^3 \quad [46]$$

$$U_{Q_{ob,1}} = 3\% \times 40\ L/\min = 1.2\ L/min = \pm\ 2 \times 10^{-5}\ m^3/s \quad [47]$$

$$\theta_{Q_{ob,1}} \times U_{Q_{ob,1}} = 4.10 \times 10^{-5}\ kg_a/s \text{ Eqn. } [48]$$

$$\theta_{P_1} = \frac{\partial \dot{m}_{a1}}{\partial P_1} = \frac{18.0272Q_{ob,1}}{v_1\sqrt{(36.0544P_1+530)(T_1+460)}} = 2.24 \times 10^{-5}\ kg_a/(s \cdot psi) \quad [49]$$

$$U_{P_1} = 2\% \times 30\, psi = \pm\, 0.6\, psi \quad [50]$$

$$\theta_{P_1} \times U_{P_1} = 1.344 \times 10^{-5}\ kg_a/s \quad [51]$$

$$\theta_{T_1} = \frac{\partial Q_1}{\partial T_1} = \frac{(-18.0272P_1 - 265)Q_{ob,1}}{(T_1+460)^2 v_1 \sqrt{\frac{36.0544P_1+530}{T_1+460}}} = 8.87 \times 10^{-7}\ kg_a/(s \cdot ℃) \quad [52]$$

$$U_{T_1} = \pm\, 0.2℃$$

$$\theta_{T_1} \times U_{T_1} = 1.774 \times 10^{-7}\ kg_a/s \quad [53]$$

$$\theta_{v_1} = \frac{\partial Q_1}{\partial v_1} = -\frac{Q_{ob,1}\sqrt{\frac{36.0544P_1+530}{T_1+460}}}{v_1{}^2} = \text{-}0.00162\ m^3/kg \quad [54]$$

$$U_{v_1} = \pm\, 2.25\%$$

$$\theta_{v_1} \times U_{v_1} = -3.64 \times 10^{-5}\ kg_a/s \quad [55]$$

$$U_{\dot{m}_{a1}} = \sqrt{\left(\theta_{Q_{ob,1}} U_{Q_{ob,1}}\right)^2 + \left(\theta_{P_1} U_{P_1}\right)^2 + (\theta_{T_1} U_{T_1})^2 + (\theta_{v_1} U_{v_1})^2} = 5.65 \times 10^{-5}\ kg_a/s \quad [56]$$

$$\dot{m}_{a1} = 9.50 \times 10^{-4}\ kg_a/s \pm 5.65 \times 10^{-5}\ kg_a/s = 6.65 \times 10^{-4}\ \ kg_a/s \pm 8.50\% \quad [57]$$

$$\theta_{Q_{ob,3}} = \frac{\partial \dot{m}_{a3}}{\partial Q_{ob,3}} = \frac{1}{v_3}\sqrt{\frac{36.0544P_3+530}{T_3+460}} = 0.61\ kg_a/m^3 \quad [58]$$

$$U_{Q_{ob,3}} = 3\% \times 40\ L/\min = 1.2\ L/min = \pm\ 2 \times 10^{-5}\ m^3/s \quad [59]$$

$$\theta_{Q_{ob,3}} \times U_{Q_{ob,3}} = 1.22 \times 10^{-5}\ kg_a/s \quad [60]$$

$$\theta_{P_3} = \frac{\partial \dot{m}_{a3}}{\partial P_3} = \frac{18.0272Q_{ob,3}}{v_3\sqrt{(36.0544P_3+530)(T_3+460)}} = 2.1 \times 10^{-5}\ kg_a/(s \cdot psi) \quad [61]$$

$$U_{P_3} = 2\% \times 30\, psi = \pm\, 0.6\, psi \quad [62]$$

$$\theta_{P_3} \times U_{P_3} = 1.26 \times 10^{-5}\ kg_a/s \quad [63]$$

$$\theta_{T_3} = \frac{\partial Q_3}{\partial T_3} = \frac{(-18.0272P_3 - 265)Q_{ob,3}}{(T_3+460)^2 v_3 \sqrt{\frac{36.0544P_3+530}{T_3+460}}} = 3.77 \times 10^{-7}\ kg_a/(s \cdot ℃) \quad [64]$$

$$U_{T_3} = \pm\, 0.2℃$$

$$\theta_{T_3} \times U_{T_3} = 7.54 \times 10^{-8}\ kg_a/s \quad [65]$$

$$\theta_{v_3} = \frac{\partial Q_3}{\partial v_3} = -\frac{Q_{ob,3}\sqrt{\frac{36.0544P_3+530}{T_3+460}}}{v_3{}^2} = 3.08 \times 10^{-4} m^3/kg \quad [66]$$

$$U_{v_3} = \pm\, 2.25\%$$

$$\theta_{v_3} \times U_{v_3} = 6.92 \times 10^{-6}\ kg_a/s \quad [67]$$

$$U_{\dot{m}_{a3}} = \sqrt{\left(\theta_{Q_{ob,3}} U_{Q_{ob,3}}\right)^2 + \left(\theta_{P_3} U_{P_3}\right)^2 + (\theta_{T_3} U_{T_3})^2 + (\theta_{v_3} U_{v_3})^2} = 1.89 \times 10^{-5}\ kg_a/s \quad [68]$$

$$\dot{m}_{a3} = 4.07 \times 10^{-4}\ kg_a/s \pm 1.89 \times 10^{-5}\ kg_a/s = 4.07 \times 10^{-4}\ kg_a/s \pm 4.64\% \quad [69]$$

2 Supplementary Figures and Tables

2.1 Supplementary Figures

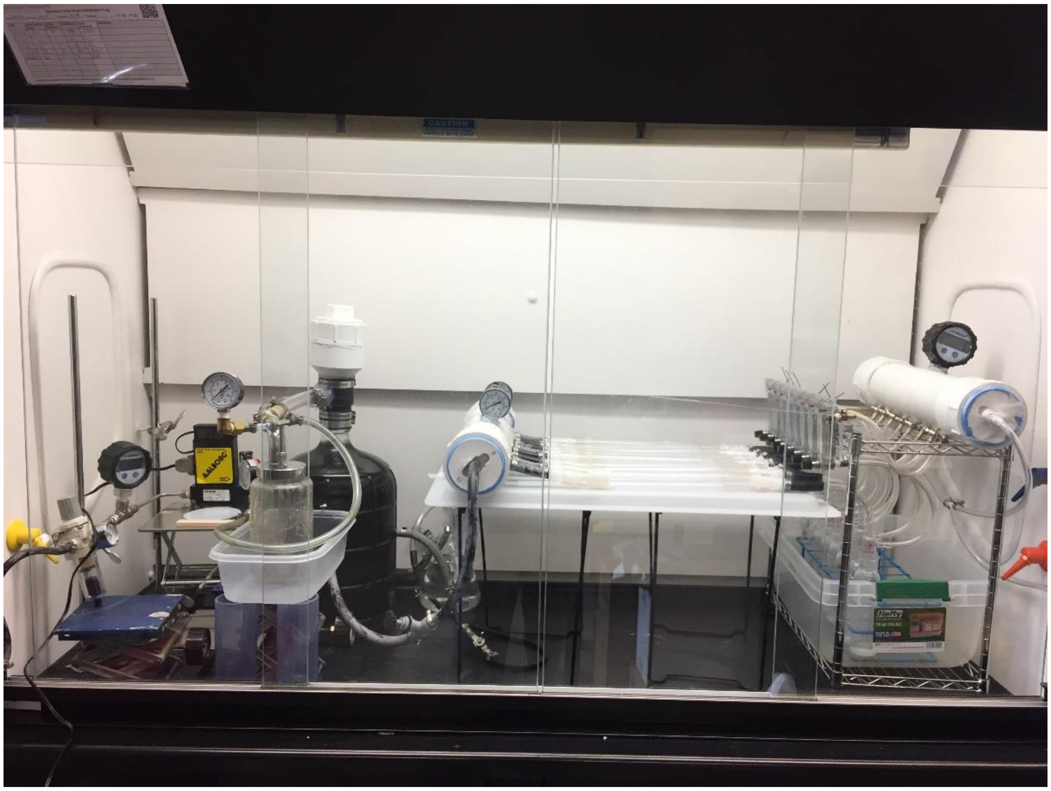

Supplementary Figure 1. Experimental setup for the generation of airborne PRRS virus and treatment with U.V. light inside a fume hood. An air compressor and a vacuum pump were located outside of the hood due to the limitation of the space. The left side air compressor was responsible for pressurizing air flowing into the system, and the right-side pump was vacuuming exhaust air coming out of the system.

Engineering considerations: PRRSV aerosolization & collection

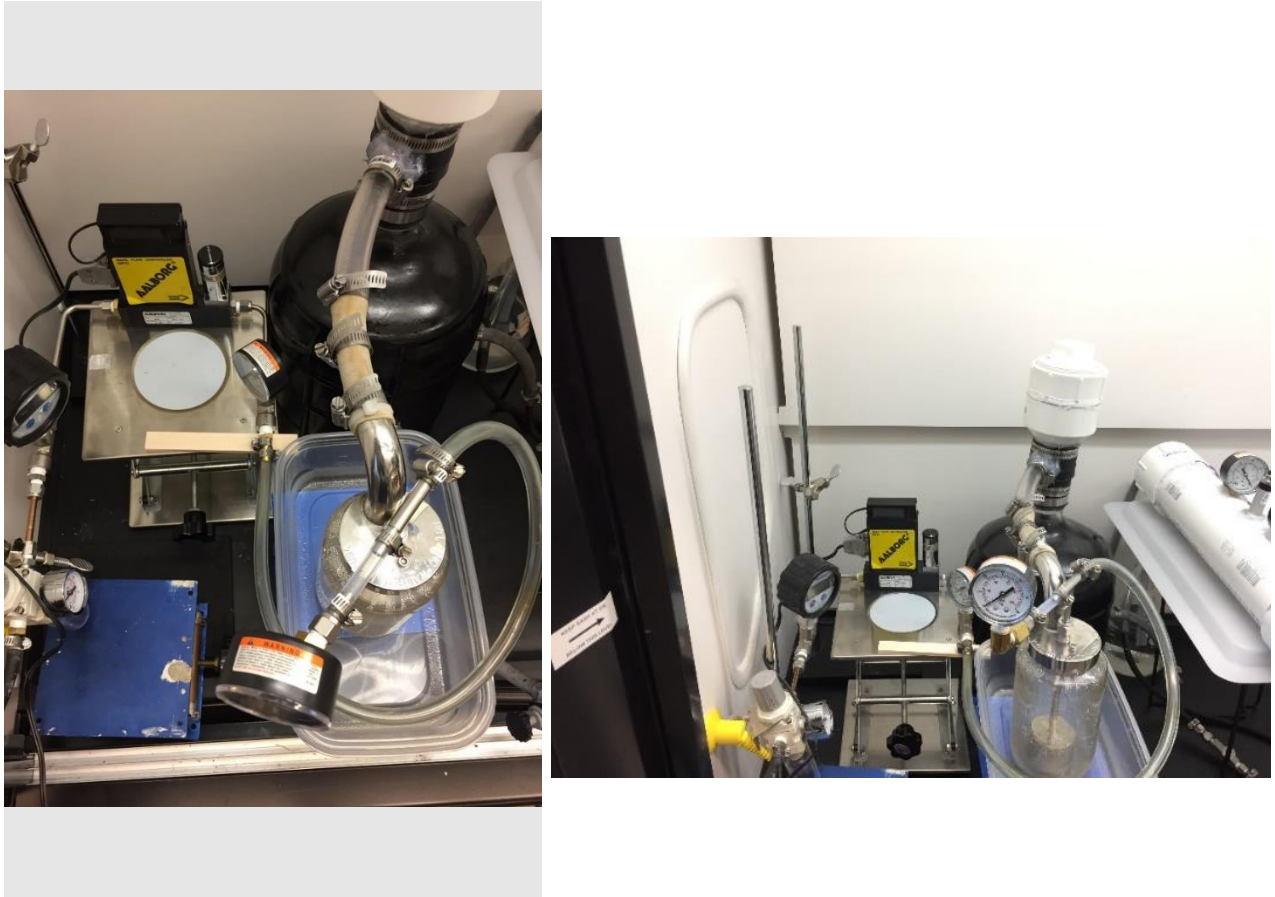

Supplementary Figure 2. View of the aerosolization section. It consists of pressure gauges and a mass flow controller, Collison nebulizer, all of which were connected by compression fittings.

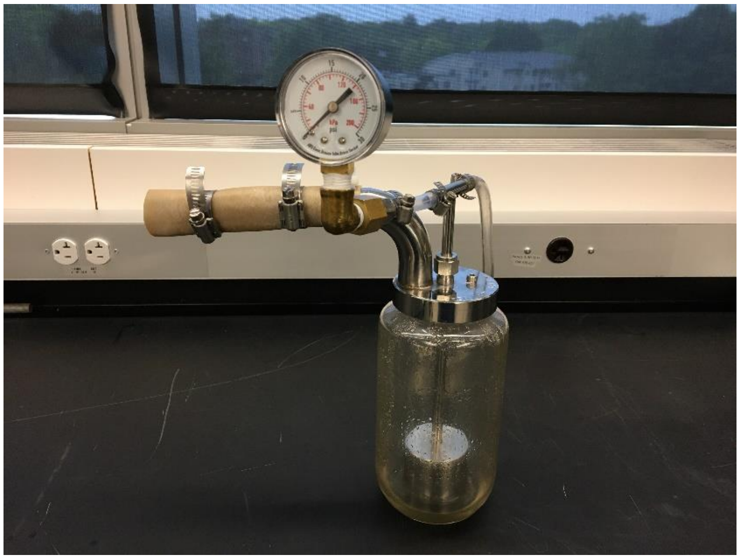

Supplementary Figure 3. A pressure gauge was installed on a 24-jet Collison nebulizer as an optional verification suggested by the manufacturer to monitor the pressure within the nebulization jar.

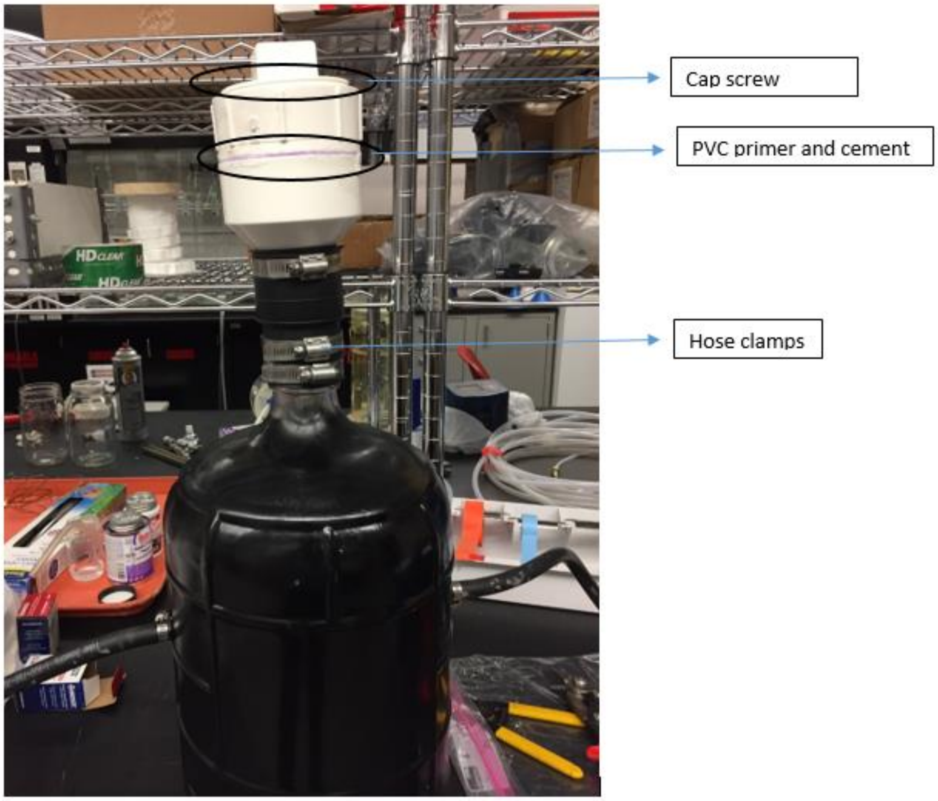

Supplementary Figure 4. The glass container was used to stabilize the aerosolized virus. A 3-gallon (12 L) glass carboy (painted in black on the outlook to prevent lighting effect) was installed with rubber hoses, PVC connectors, and hose clamps. PVC primer and cement were used to seal the gaps.

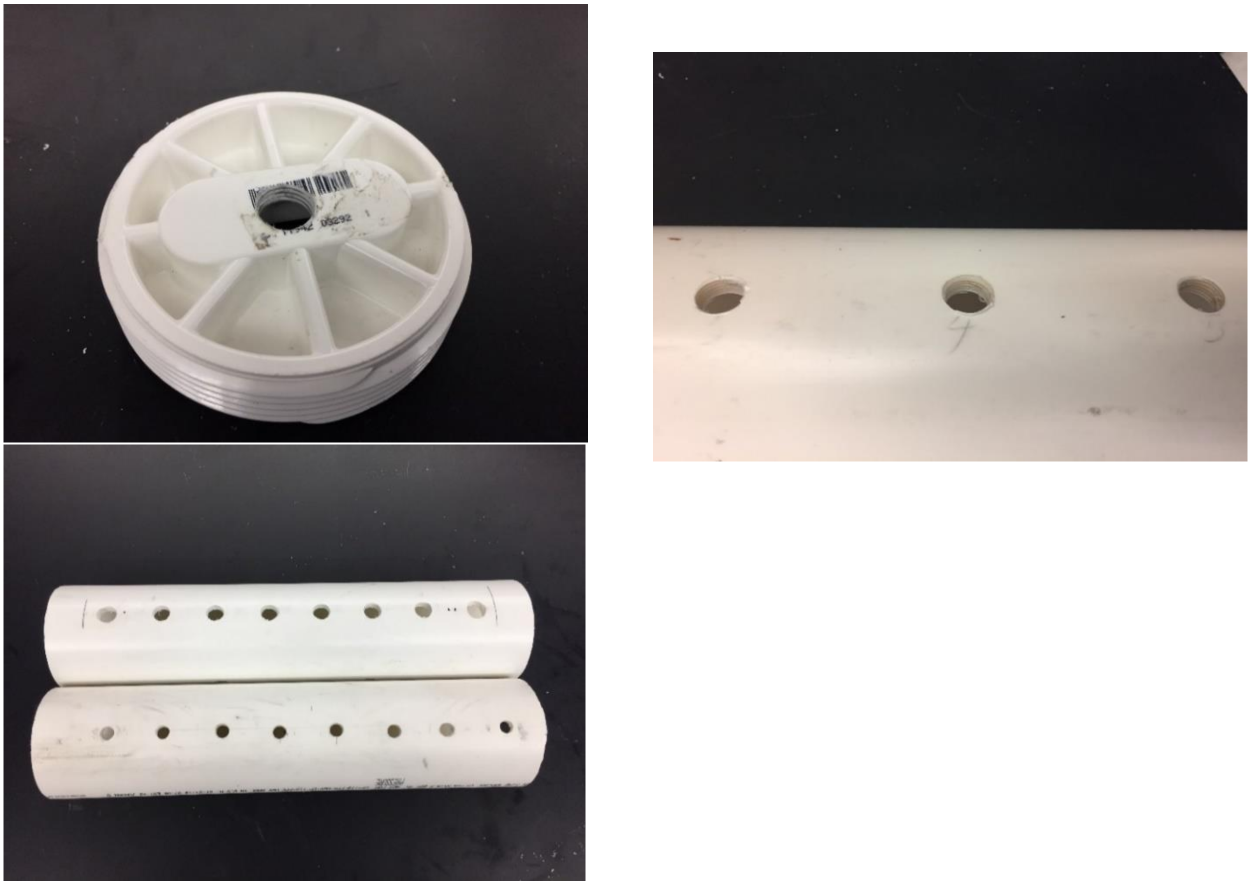

Supplementary Figure 5. Details of the construction of the manifold system for distribution of aerosolized virus into separate treatments (I). Threaded holes were used to mount brass fittings into Manifolds (1 and 2) (PVC pipes, ID = 3 in) to ensure a tight fit and lower the risk of leakage. All drilled holes were threaded.

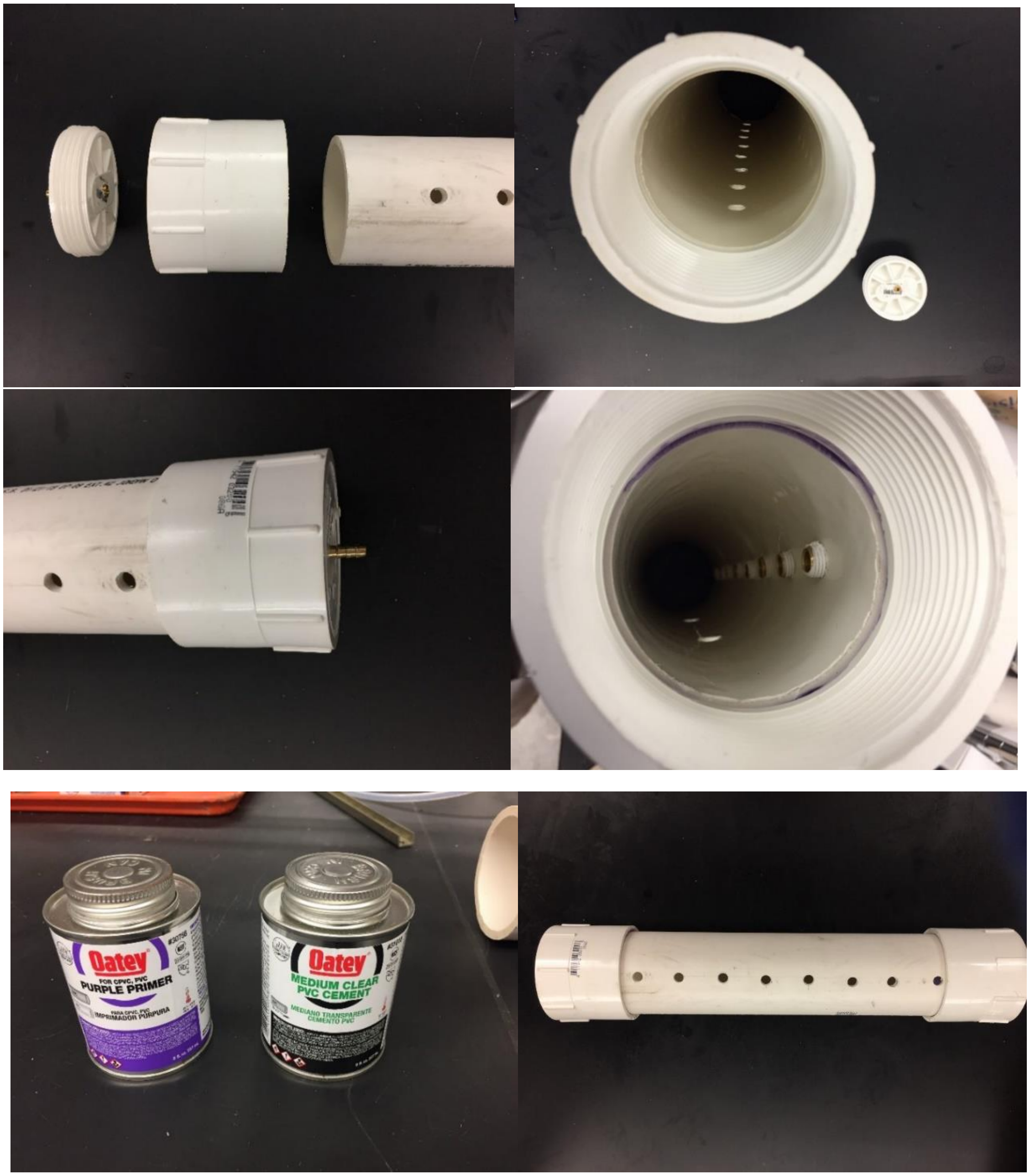

Supplementary Figure 6. Details of the manifold system for distribution of aerosolized virus into separate treatments (II). PVC adapter (ID = 3.5 in or 89 mm) and screw caps (ID = 3.5 in or 89 mm) were used to close both ends of Manifolds (1 and 2). PVC primer and cement were used to seal the gaps between these parts.

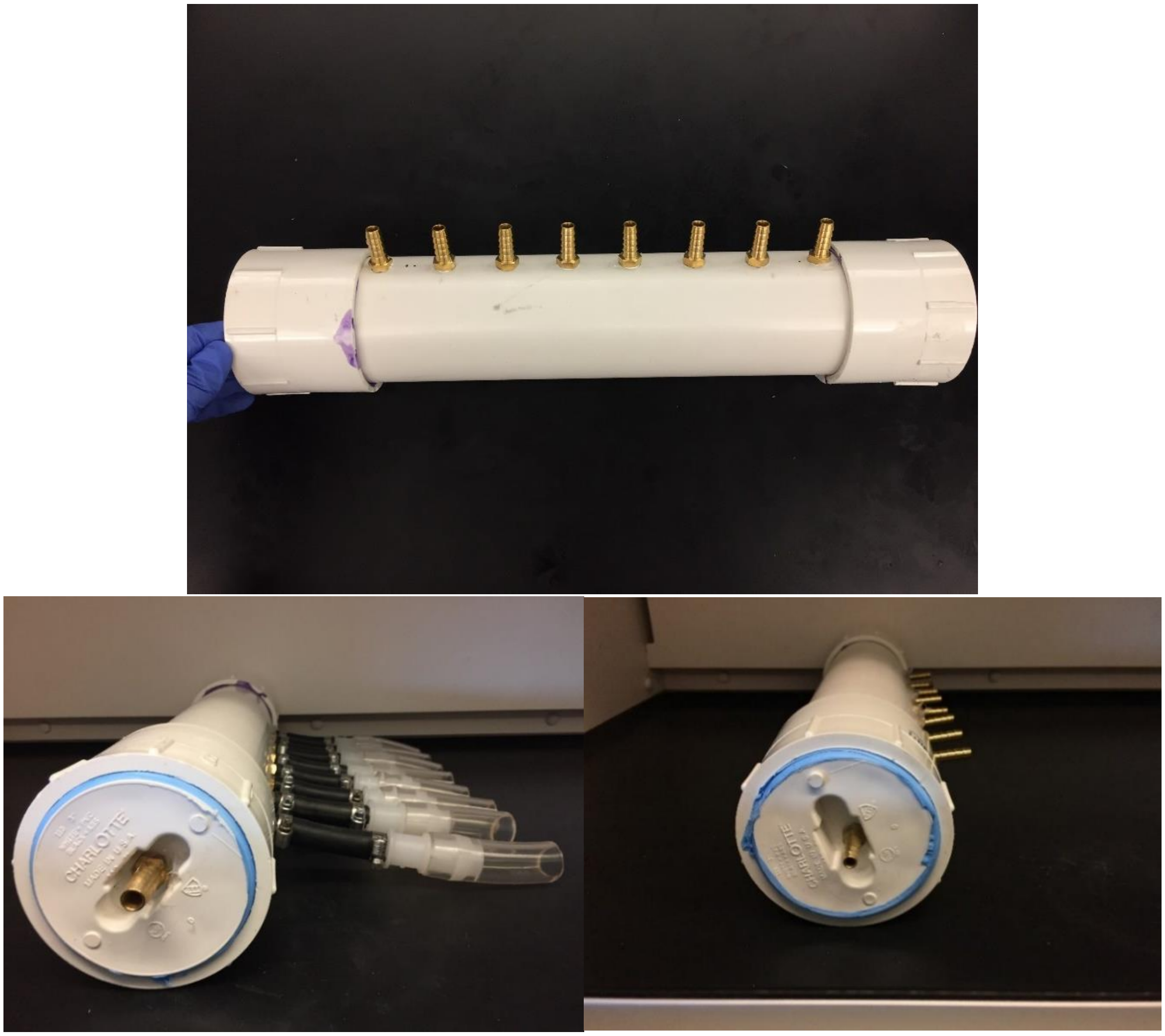

Supplementary Figure 7. Details of the manifold system for distribution of aerosolized virus into separate treatments (III). Two types of barbed hose fittings, 9.52 mm (3/8 in) and 6.35 mm (1/4 in), were installed on Manifold 1 (left) and 2 (right), respectively. Threaded fittings are used to ensure a tight fit and lower the risk of leakage.

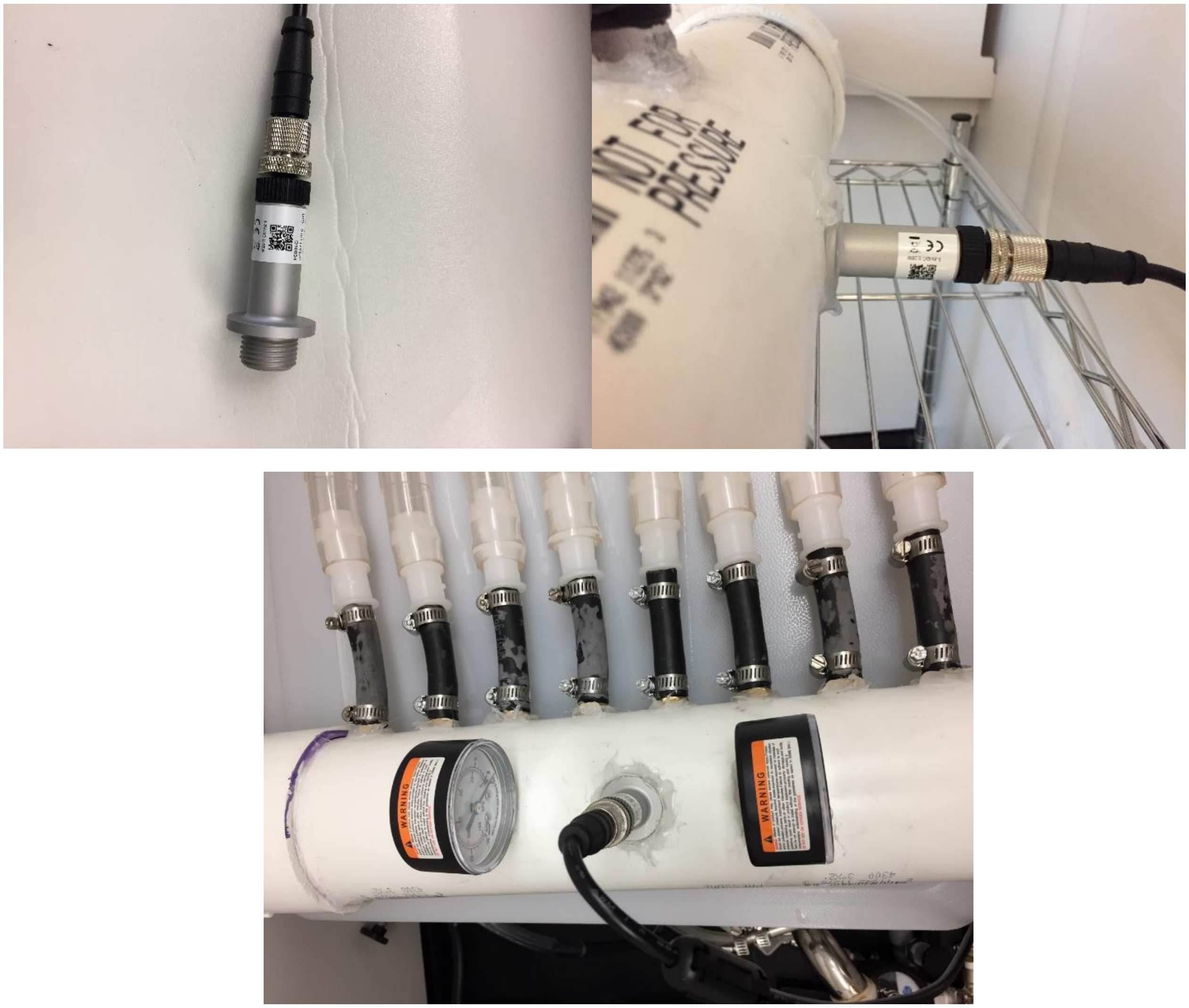

Supplementary Figure 8. Smart temperature and humidity probe (SP-004-4, Omega Engineering Inc., Stamford, CT 06907) and corresponding USB cords (IF-001, Omega Engineering Inc., Stamford, CT 06907) were mounted on both Manifolds (1 and 2) to measure temperature and relative humidity in the Manifolds. The heads of the probes were threaded by the manufacturer so they could fit in the holes and threads that were drilled on the Manifolds. A pressure gauge (0 – 30 psi) and vacuum gauge (0 - 30 in Hg) were installed for Manifold 1.

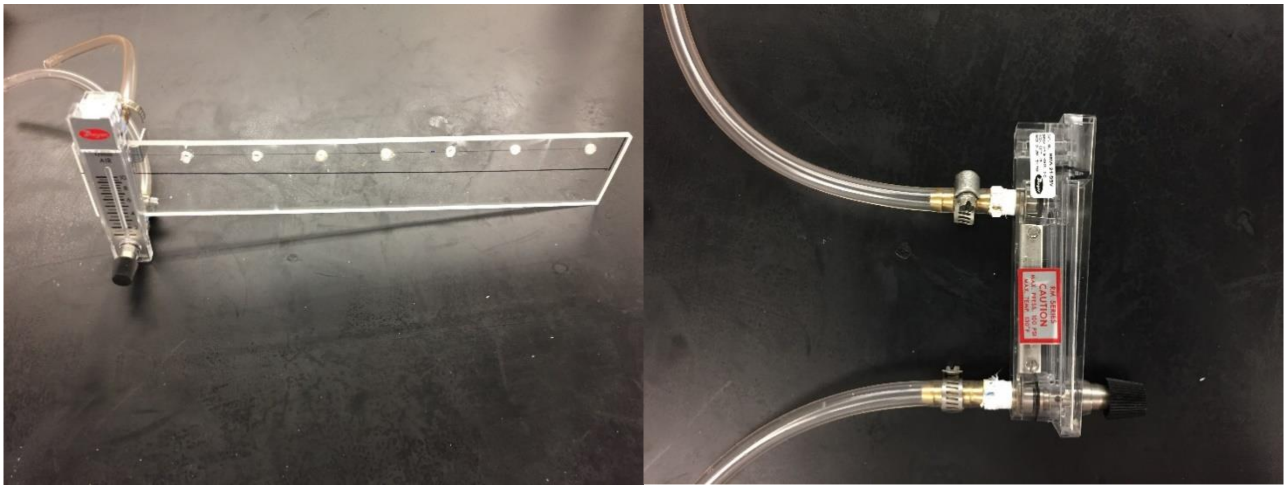

Supplementary Figure 9. Visual verification of air flowrate in each treatment (I). An acrylic board was cut, and eight holes were drilled on it so that the rotameters (RMA-21-SSV, Dywer Inc. Michigan City, IN, USA) can be mounted on it.

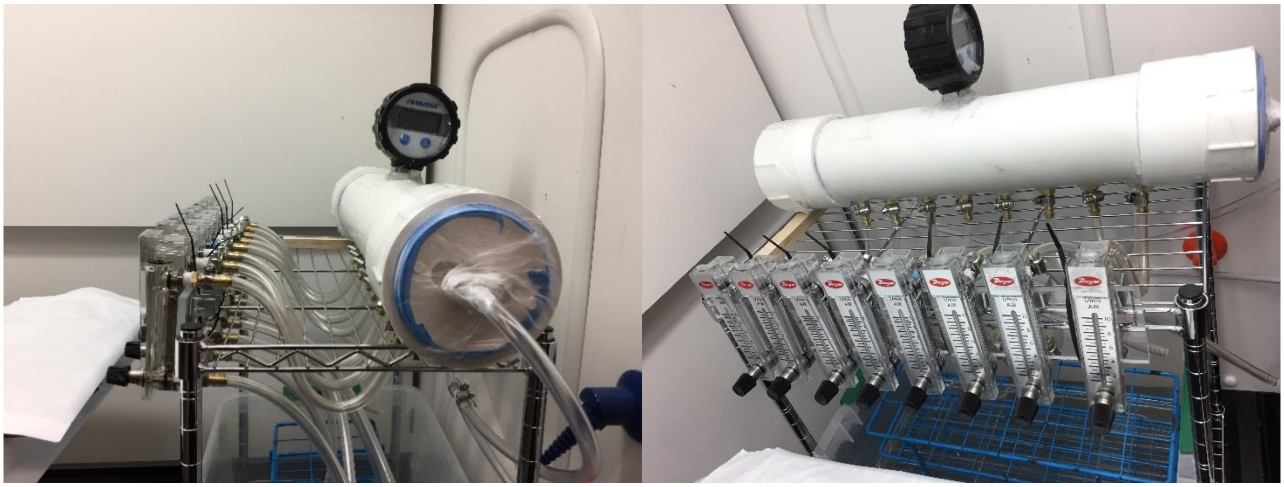

Supplementary Figure 10. Visual verification of air flowrate in each treatment (II). Eight Dwyer flowmeters were mounted on a stainless-steel shelf so that they can be positioned vertically to ensure accurate readings. A digital pressure gauge (range –15 psi to 15 psi, ±0.5% full scale) (DPG108-015CG, Omega Engineering Inc., Stamford, CT 06907) was installed on top of Manifold 2. The same installation procedure was followed as for other sensors and gauges.

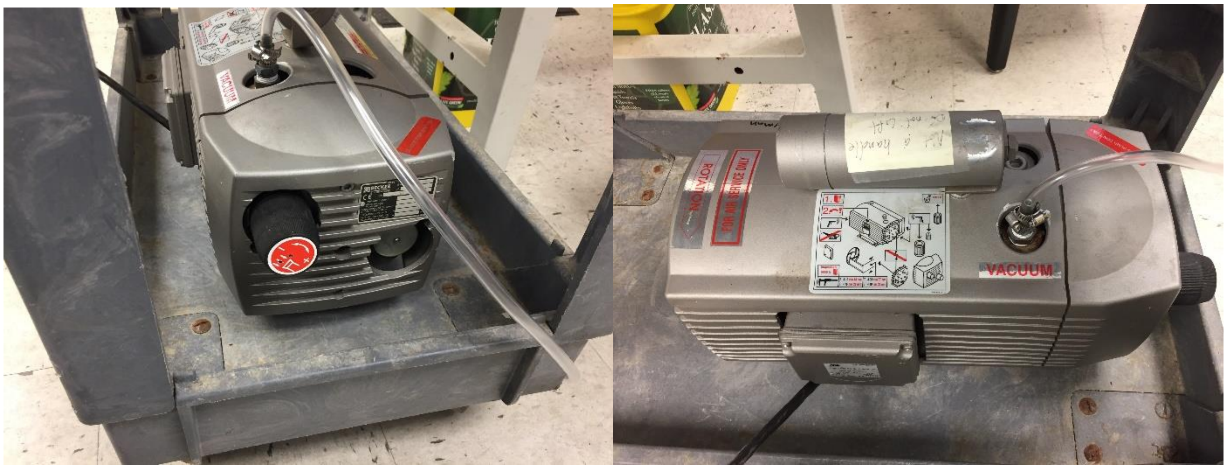

Supplementary Figure 11. Details of the air handling system. An air vacuum pump (VT 4.16 rotary vane vacuum pump, Becker Pumps Corp., Cuyahoga Falls, OH, USA) that sucks air from the outlet of the system to drive the sampling process in the impingers.
